# Measuring Information Security Performance with 10 by 10 Model for Holistic State Evaluation

**DOI:** 10.1371/journal.pone.0163050

**Published:** 2016-09-21

**Authors:** Igor Bernik, Kaja Prislan

**Affiliations:** University of Maribor, Faculty of Criminal Justice and Security, Kotnikova 8, SI - 1000, Ljubljana, Slovenia; West Virginia University, UNITED STATES

## Abstract

Organizations should measure their information security performance if they wish to take the right decisions and develop it in line with their security needs. Since the measurement of information security is generally underdeveloped in practice and many organizations find the existing recommendations too complex, the paper presents a solution in the form of a 10 by 10 information security performance measurement model. The model—ISP 10×10M is composed of ten critical success factors, 100 key performance indicators and 6 performance levels. Its content was devised on the basis of findings presented in the current research studies and standards, while its structure results from an empirical research conducted among information security professionals from Slovenia. Results of the study show that a high level of information security performance is mostly dependent on measures aimed at managing information risks, employees and information sources, while formal and environmental factors have a lesser impact. Experts believe that information security should evolve systematically, where it’s recommended that beginning steps include technical, logical and physical security controls, while advanced activities should relate predominantly strategic management activities. By applying the proposed model, organizations are able to determine the actual level of information security performance based on the weighted indexing technique. In this manner they identify the measures they ought to develop in order to improve the current situation. The ISP 10×10M is a useful tool for conducting internal system evaluations and decision-making. It may also be applied to a larger sample of organizations in order to determine the general state-of-play for research purposes.

## Introduction

The contemporary business environment is characterized by unpredictable changes, pressures, aggressive competition, informatization and global connectivity. Its normal functioning is becoming ever more dependent on complex information systems and advanced technologies. The success of organizations operating in such an environment is also strongly related to the adequate decision-making in the field of information security management. Cyber threats are causing major disruptions and enormous financial damage in the corporate environment at the global economic and security level. This is why security agendas of international organizations, such as European Union, NATO and World Economic Forum, place cyber threats among the most important work priorities and global security-related issues [1, 2, 3]. Organizations whose processes and development rely heavily on IT are exposed to unprecedented risks posed by cyber threats and, more than ever, in need of a strong information security system.

When discussing the management of business information systems, information security is undoubtedly an essential requirement for existing or trending technologies (such as IoT or Cloud services) [4, 5]. Information security means that data or information cannot be accessed in an unauthorized manner for the purpose of their use, disclosure, interruption, change, etc. [5]. Any disclosure of sensitive information, particularly personal data or intellectual property, is unacceptable [4], which is why security measures are needed to prevent and/or detect technical abnormalities and user misbehavior that could lead to information incidents [6].

In terms of its characteristics, information security is most commonly related to the well-known CIA triad [7] that emphasizes the importance of confidentiality, integrity and availability of information and related information sources. In this regard, trustworthiness is one of the most critical aspects, since the extent of data reliability and trustworthiness is of utter importance for achieving high performance and efficient decision-making [6]. Apart from the aforementioned criteria, other authors emphasize additional critical principles, such as authentication, privacy, non-repudiation, access control and availability [5, 6].

Researchers in the information security profession point out that corporate information security must be developed as a strong business function and not simply as an exclusively technical activity [8, 9, 10, 11, 12, 13, 14]. The future of an entire enterprise namely depends on the quality of security and risk management—information, information systems and technologies may bring about numerous benefits to any organization, however, they can also become its main source of vulnerability if they are not managed efficiently. It is not just a question whether organizations have any security measures in place, but there is a wider issue concerning information security planning. If information system planning is not properly aligned with business strategy and planning this may lead to lower overall business performance [15].

Information security is one of the major managerial challenges, as it deals with the management of a complex system where wrong decisions may lead to poorer performance of the entire organization [16]. When an organization is competent and capable of providing a high level of security, it is also efficient with respect to its planning, organization, implementation and investment activities [8]. The overall performance of an organization is therefore dependent on the alignment between information system (and security) planning and business planning [15], which is often difficult to achieve, particularly when considering the paradoxical situation that lingers between the needs and wants of an organization: it *needs* a strong security system in order to minimize risks and *wants* a high level of functionality and availability of those secured information systems to achieve its productivity goals.

Similarly to any other security activities, measures related to information security also limit some of the critical and much needed functionalities of (business) information systems [4], thus creating a necessity to plan restrictions in a rational and analytical manner. When planning information security, one needs to consider that new technologies are being implemented for improving the ability to make quick and correct decisions and increasing the availability of required critical information in a mobile manner [17]. Remote access and interoperability are also among such requirements [5]. Following these requirements, an approach to building an effective business information system demands multidisciplinary skills and cross-disciplinary collaboration [17], while business models need to meet criteria related to cooperative control, agility, flexibility, operability and robustness [18]. In this way, the risk of narrow-minded and biased decisions is reduced, while plans are more adaptive and applicable to different situations.

Finally, efficient information security management requires numerous professional skills and a systematically organized approach, which organizations often do not have. Reports on organizational practices show that more than one third of organizations observe below average and insufficient results when attempting to guarantee information security, while this area is adequately managed by less than one quarter of all organizations [19, 20, 21]. Apart from alarming predictions about the future development of information threats, there is an even more distressing finding: the majority of information threats currently faced by organizations are elementary in their nature. Reports show that nearly 75 per cent of incidents could be easily prevented by basic tools, which means that threats generally exploit well-known vulnerabilities, which organizations are unable to manage properly [22. 23]. In addition, organizations also lack capabilities related to the threat and vulnerability detection area. For example, studies show that many incidents are only detected several months after they take place and are most often discovered by third parties and not by organizations themselves [24]. Although this may not seem problematic, considering that the least sophisticated threats can still bypass existing security controls, it actually demonstrates organizations’ unpreparedness for emerging targeted threats. The stagnation of information security is problematic because the simplest types of incidents may actually cause major consequences, which will become even greater in the future.

Available data regarding the state-of-play in the field of information security demonstrate that the management of this field is generally inadequate and full of poor practices [19, 20, 21, 25, 26]. The reasons for this may be found in the fact that a large number of actual information incidents is directly related to unfavorable economic conditions, which creates particularly paradoxical and difficult circumstances. Organizations require a high level of information security if they wish to avoid severe consequences, but at the same time, they need to rationalize resources dedicated to their management. It is therefore necessary to guarantee a successful information security performance with minimum investments, while the management has to know how to decrease costs and simultaneously improve or increase the cost-performance ratio [27]. This cannot be achieved if organizations are not familiar with their baseline situation and do not know which goals they wish to achieve.

When organizations are trying to establish their information security systems they are facing different and interrelated obstacles, such as the lack of financial means, knowledge and competences, which currently represents the main issue. For example, organizations may have established a set of guidelines and adopted security plans, but in reality, they find it difficult to implement all appropriate measures to achieve compliance. This happens due to the lack of financial means, which is also generally the greatest obstacle to information security [28, 29, 30]. Another obstacle, which often appears in parallel to inadequate financial support, is related to inappropriate attitudes of the management and their lack of awareness, which contribute to the preservation of an extremely traditional, technically oriented attitude towards information security [31]. The security of less developed organizations is often dominated by the view that information security is the sole responsibility of IT departments, while responsibilities in the field of technology, security and privacy are usually assigned to a single person. The final performance of the entire security system thus often depends on the competences and abilities of that (overburdened) person [32, 33]. This is particularly problematic if one takes into account that enterprises are commonly overly self-confident and excessively optimistic when assessing their own vulnerability and ability to manage such situations [8]. Apart from the aforementioned shortcomings, it is also possible to observe a certain level of indifference with respect to information security risks, as organizations tend to be overly confident about their system’s abilities or erroneously convinced that they cannot be the perpetrators’ primary target.

Adequate and economical decisions can only be taken when organizations measure their information security performance and monitor the effectiveness of implemented measures. Information security performance measurement is a tool that may be used by the management to support their decisions. However, experts and research studies point out that in practice such procedures are underdeveloped and deficient, while security measures do not comply with actual security needs [11, 20, 23, 34]. This is why it is difficult for the management, which lacks competence in the field of information security, to recognize the most efficient measures in the abundance of technologies, vulnerabilities and potential solutions [14, 35]. When one considers the exponential development of the threat environment and increased pressures exerted upon organizations by the market, it becomes clear that they require fast, innovative and practical solutions, which will enable them to perform a solid analysis of their security needs and obtain a general assessment of the state-of-play in the field of information security.

In fact, organizations may rely on existing standards and information security models, which are of high quality, however, some organizations find them only conditionally applicable in practice. When considering the capabilities of an average organization facing unfavorable economic conditions, certain recommendations are excessively complex and demanding in terms of required expertise and financial means. They also lack transparency and are too dispersed. There are over one thousand different standards and handbooks, and the abundance of recommendations makes it difficult for organizations to choose the appropriate ones. Experts from The UK Department of Business Innovation and Skills conducted a review of 128 most important standards related to information security and found that they lack a holistic approach. Standards mostly focus on a particular aspect while ignoring other equally important elements. When considering the excessively complex nature of recommendations and the fact that organizations often lack financial resources to follow such standards, it becomes clear that many organizations are forced to rely on their own limited capabilities for measuring and planning information security [36]. They may hire external consultant groups to perform analyses and assess the performance of processes, but it is generally recommended that organizations perform in-house assessments and gain their own experience in conducting assessment procedures [30, 34]. The proposed model ISP 10×10M allows organizations to conduct their own information security assessments. Its form and methodology were created by considering the most important recommendations regarding the design of measuring instruments.

The goal of this paper is to present a tool enabling a simple and practical information security assessments that will allow management make plans for next-steps. The main application of ISP 10×10M in organizations represents the basis for assessing the general level of information security in a given business environment. Since the model is universal and is functioning according to the principles of index measurements, we can also use it to assess and categorize different organizations into levels of information security performance. Therefore, we can use it as a case study tool or research instrument for wider comparable study.

The paper is organized as follows: the next three sub-sections review information security measurement approaches, recommendations in organizational security performance measurement and theoretical background of our key performance indicators. Section 2 describes methodology and the process of instrument and model development. Section 3 outlines the ISP 10×10M and its evaluation by information security experts, the weighting process and performance measurement solutions that contain six information security levels. Section 4 discusses research results, the potential use of the model in practice and its limitations. The paper ends with conclusions and suggestions for further work.

### Measuring Information Security

By measuring information security performance, organizations determine the extent to which their security needs are met. Such measurements may apply to the entire system or to its individual elements. Apart from systems’ performance, organizational, behavioral and criminological aspects or other narrower operational, programming and technical fields may also be examined. In general, techniques used to measure information security performance are divided into quantitative and qualitative categories [14, 37], while a combination of approaches is also possible.

Quantitative information security analyses represent a traditional approach to measurement. They include [9, 14, 27, 30, 38]:

- technical analyses and testing of information systems—e.g. penetration testing,
- mathematical models or statistical computations of risks—e.g. risk analysis and
- economic analyses of investment justification—e.g. ROI.

Such analyses enable organizations to determine whether perpetrators would be able to circumvent security controls, how likely it is for certain threats to materialize and whether investments into specific measures will pay off. The advantages of such analyses, provided they are conducted properly, can be found in their accuracy and reliability, but their individual procedures are very complex and require a great deal of time and knowledge. In addition, such analyses must be performed in combination with other types of analyses, as it is vital to first identify individual vulnerabilities or the scope of investments. Since security is a complex system, which makes it impossible to anticipate all threats and vulnerabilities, quantitative analyses are somewhat incomplete and insufficient when considered in isolation, despite their accuracy [9]. Hence, some authors [11, 35, 39] point out ever more frequently that economically oriented models are too biased, since the profitability of measures cannot be the only performance criterion in information security. Nevertheless, technical and economic models should not be denied their validity, particularly when considering that measures implemented at operational levels represent the most common and most developed approach to providing information security. However, one must be aware that adopting technical and operational measures alone is insufficient, since the performance of organizations in the era of complex information security issues also depends on other tactical and strategic aspects [8, 32, 40, 41].

The ever-greater amount of attention paid to management, culture, policies and users relationships aimed at clarifying issues related to information security is improving our knowledge and awareness of its complex nature. It also influences measurement models, which are ever more frequently becoming of qualitative nature and comprise systematic assessments of predetermined criteria with a view to determine the level of security strategies’ implementation [14]. In addition to classic information security controls (or risks and compliance) check-lists, qualitative models also include approaches that analyze information security from socio-psychosocial, organizational and criminological point of view. These newer and contemporary approaches are examined by an ever-greater number of experts (e.g. [8, 10, 40, 42, 43, 44]). In this context, information security models rely on following theories:

- Behavioral: rational choice theory, theory of planned behavior and protection motivation theory (e.g. [40, 41, 45]).
- Organizational: TOE framework, organizational complexity, performance, strategy and culture (e.g. [33, 35, 46, 47]).
- Criminological: general deterrence theory, situational crime prevention theory (e.g. [13, 27, 48, 49]).

These explain how organizational and personality factors, social contexts, organizational culture, interpersonal relationships, external environments, preventive and response measures and sanctions influence users’ behavior and consequently contribute to information systems’ security. Despite the greater breadth of their scope, qualitative methods also contain several weaknesses. Research studies are often focusing on measuring isolated elements and inter-connections, which is why their findings have a narrow scope of practical application. In addition, similarly to quantitative models, qualitative methods are also insufficient, as they cannot accurately measure all areas, identify all threats and harmonize the often diverging views or needs of experts and users. The results of such analyses may be extremely subjective or relative, particularly when individual areas are assessed on the basis of intuition or biased logic [34, 40, 50].

In light of the above, it is clear that all methods and techniques have their own strengths and weaknesses. It would be ideal if organizations would assess their information security at different levels by applying a combination of qualitative and quantitative methods, thus achieving the most reliable results. Experts [31, 51] for instance, state that information security needs to be simultaneously analyzed from financial, organizational, normative, cultural, technical, procedural and risk management aspects. Naturally, such an approach is very advisable, but often too demanding and unfeasible due to organizations’ constraints. Combined approaches and quantitative analyses are primarily recommended for larger organizations, where the complexity of systems and quantity of resources are greater, while smaller organizations are advised to mainly apply qualitative system and process analyses, as these are easier to use and generate results rather quickly [9, 35, 37].

Despite the abundance of models and recommendations used for assessing information security performance, many authors (e.g. [11, 16, 34, 46]) point to the lack of studies that would measure or consider information security in a comprehensive manner by applying specific positioning indicators. Existing models are criticized for their narrow focus and excessive theoretical tendencies or impracticability. However, it has to be stressed that the purpose of this paper is not to criticize or ignore existing models and research studies, but identify the advantages of individual approaches and bridge existing gaps in this field. It also aspires to provide organizations with a faster and more holistic solution by analyzing endeavors made in this field thus far.

### Key Performance Indicators—Theoretical Background

The proposed model ISP 10×10M falls in the scope of qualitative assessment of organizations’ systems. Over twenty years ago, Kaplan and Norton [52] developed a method for assessing organizational performance for the purpose of strategic planning (i.e. balanced scorecards), which is still used today. As they state in their recommendations, a preliminary identification of areas that define the performance of a system subject to measurement (i.e. critical success factors [CSFs]) is a precondition for successful measurement. Apart from critical success factors, it is also necessary to determine criteria (i.e. key performance indicators [KPIs]) used to measure the extent to which an organization is successful in regulating an individual area or a systemic factor. Background, which helped us to establish a good and solid model with a list of relevant factors and indicators, is presented in the following paragraphs. When developing the ISP 10×10M, we considered the fact that organizations primarily require simple and easily applicable procedures to establish their baseline.

Since many existing models are labeled for having narrow scope of application, the first condition taken into account when developing ISP 10×10M refers to multi-dimensionality and the fact that individual parts of organizational systems cannot be considered in isolation when assessing information security performance. Information security reflects the state-of-play of an entire organization, it touches upon all business processes and all aspects on an organization’s life, which is why it requires a team approach, cooperation and links to other organizational functions [53]. Apart from technical and logical repressive measures, organizations must also develop capabilities at user, procedural, policy and organizational levels [47, 52]. When designing the content of ISP 10×10M, we relied on the so-called TOE framework (Technology, Organization, Environment) proposed by Tornatzky and Fleischer [54]. This framework presupposes that organizations’ technological development needs to consider three main perspectives: the technological context, which comprises internal processes, assets and technology; the organizational context, which involves organizations’ vision, strategy, goals and structure; and the environmental context, which includes factors, such as business environment and circumstances in an industry, competition, legislation and politics.

The multi-dimensional approach is closely related to the condition requiring adequate planning and management of information security. Information security cannot be effective without strong managerial support and competent security management [34]. In this context, strategic planning, which manifests itself in the form of adopting a strategy and providing adequate resources for the implementation of plans and decisions, is particularly important [41, 52]. As explained previously, sound decisions and plans can only be adopted on the basis of the right information, which organizations obtain through continuous control and assessment procedures [10, 32]. Information security performance measurement is therefore an important process within information security planning, which falls under the management’s responsibility and most definitely represents one of the main performance criteria.

Since threats to information assets are heterogeneous and stem from various causes, the setting of criteria is based on a premise requiring that measures be implemented at different levels [41, 55]. This is also related to the need for an in-depth and architectural protection, which means that vulnerabilities must be examined in a multifaceted manner [39, 56]. If one considers criminological and behavioral theories, it becomes clear that perpetrators take their decisions based on weighting risks and benefits. When the probability of detection is high and followed by serious sanctions, and the commission of an act requires a great deal of effort, the likelihood of undesirable behavior is lower [48]. Such likelihood is also lower when organizations provide an adequate balance between preventive and responsive measures [13, 47]. Preventive activities are important up to the moment in which an incident takes place; they include both situational as well as socio-preventive measures. In this context, situational prevention refers to measures for preventing or making an undesirable act more difficult (e.g. detection, decreasing the appeal of potential targets, concealing assets, multi-layer protection, etc.), while social prevention comprises measures related to the management of culture and policies (e.g. creating a suitable atmosphere among users, increasing motivation, social influence and control, formal rules, etc.). When an incident takes place, preventive capabilities must be replaced by responsive capabilities, which govern the way in which organizations contain and manage an attack or violation. Responsive activities include measures in the field of crisis management, recovery, damage limitation and business continuity.

Compliance and policy also represent important elements of information security performance [10, 42, 46], without which organizations cannot formally prove their security responsibility. In order for organizations to appear responsible and win the trust of their customers or potential partners, they need to prove their responsibility through ethical behavior and transparent business operations, which respect formal and objective criteria. There are numerous recommendations for the adoption of an appropriate policy; however, it is primarily important for such a policy to exist, for users to be familiar with it, and for it to be clear in areas, such as risks, responsibilities, competences, controls and sanctions. Apart from internal formal criteria, organizations must also meet external normative requirements imposed by the State, partner or international organizations [32]. In this context, the principles of legality, proportionality and professional competence, as well as the respect of privacy and adequate protection of personal data, are particularly important.

The next important element refers to behavioral and user-related aspects, since the applicability of various measures in practice depends on the level of their maturity. This is also demonstrated by a vast amount of references proving that these two areas have been subject to intense research endeavors in the past few years. In their research studies, different authors for instance, found that information security performance mainly depends on the security culture and behavior of employees [33, 40, 55]. Others complement the above findings by stating that employees’ personal judgments, sense of self-control, knowledge and competences, as well as social relationships affect the level of compliance with security rules and policies [41, 49]. Due to its impact on the course of various processes, security culture management, which is implemented by increasing employees’ motivation, providing guidance to their work and awareness-raising activities, must represent an integral part of comprehensive information security management. The social aspect is also closely linked to the legitimacy of information security, which is related to the institutional theory. The latter presumes that apart from third party relationships, security culture and the external environment, organizations’ security operations are also influenced by the legitimacy of planned activities and regulating controls [57]. If information security is to become generally accepted by users or legitimate, it must have a minimum impact on the course of work processes and users’ productivity [42]. Studies show that the occurrence of errors and deviations is greater when measures are overly repressive, as users are not willing to sacrifice their productivity at the workplace to information security [39, 40].

Although the next perspective is less frequently discussed in scientific circles, it was included into ISP 10×10M due to its influence on information security performance. It involves impacts and factors from the external environment that cannot always be managed by organizations. The nature of such impacts is extremely diverse, but in principle, all organizations are facing pressures from their external environment, which may represent an obstacle and require adjustments of organizational processes. Such impacts include, for instance, the involvement of third parties (e.g. partners, suppliers, customers, contractors), changes in legislation and policies (e.g. pressures, lobbying, tightening of rules and regulations), changes in the environment in which enterprises operate (e.g. expansion to new markets with different regulatory and cultural rules), changes in business, security, technical and technological fields (e.g. emergence of new threats or vulnerabilities, new services and technologies, changes in supply and demand) [11, 35]. From information security point this means that neglecting external elements leads to degradation of performance, which is why its redevelopment and continuous upgrading are of vital importance. When applying this criterion, organizations must also understand that information security performance worsens in time. Organizational systems can remain viable only if they constantly develop. Information systems must be flexible, while their technological and process-related features must adapt to market requirements and demands of the local environment in which they operate.

The above-described analysis of different information security related areas, clearly shows that information security cannot be provided in an unplanned and unsystematic manner, as the scope of individual conditions and criteria is too broad. In order to develop a structure of the proposed model that would be as organized and accurate in terms of its content and processes as possible, the aforementioned theoretical and scientific premises were supported by a review of important standards and models (see S1 Table). We believe that it was reasonable to consider such standards, as they contain examples of best practices and high quality guidelines. In doing so, we were interested in determining which measures were advisable and which areas were deemed most important for establishing and managing information security. Such a descriptive method was the basis for developing the model’s content. Consequently, individual elements of the ISP 10×10M are based on scientific research studies, models or information security standards. Findings obtained through the analysis of standards and models are presented in S1 Table.

The aforementioned conditions represent the foundation of our ISP 10×10M for ensuring an efficient and high quality information security. In summary, information security is efficient when:

- physical security and adequate maintenance of information and technological infrastructure are provided,
- operational (technical, logical) measures aimed at preventing violations related to the use and operation of systems are being implemented,
- all information sources are identified and the ways of their permissible use are prescribed,
- users are qualified, aware, vetted and under control,
- analyses of information risks and business continuity plans are being implemented,
- it is legitimate and contributes to the fulfillment of organizational strategies,
- it is compliant with relevant legislation and an explicit information security policy is adopted,
- security management is present and competent for adopting the right decisions,
- third-party processes and relationships are formally regulated and abide by the ethical operation principle,
- organizations adapt to changes in their external environment and follow security trends.

Based on the above-described conditions, it is clear that the level of information security performance is high only when individual measures are simultaneously implemented at different organizational levels. Existing standards and models also consider information security as a system composed of measures implemented at the level of technical, organizational, user-related, political, normative, and environmental processes.

## Methodology—Development of a ISP 10×10M

The following sections present model ISP 10×10M composed of different areas and criteria used to measure internal and external organizational factors that influence information security, as well as organizations’ preventive and responsive capabilities. Since threats to information security are dynamic and constantly changing, they are extremely difficult to predict. This is why the model focuses on measuring organizations’ security capabilities and the general performance of their systems, and not on actual information threats. The ISP 10×10M was devised by conducting the following research phases:

- During the first phase, a review of existing research studies, models and standards was conducted. Its findings were used to design the model’s content, i.e. CSFs and KPIs.
- The second phase was dedicated to the testing of the model and its content by conducting an empirical study among experts dealing with information security. This step was aimed at verifying and confirming the validity and reliability of the model. The obtained evaluations served as a basis for categorizing and weighting individual factors and their indicators in terms of their importance.
- During the last (third) phase, collected data, as well as weighted and categorized indicators were used to establish the final structure of the model, define its categories, identify critical limits and develop a method for calculating information security performance.

**Fig 1:**
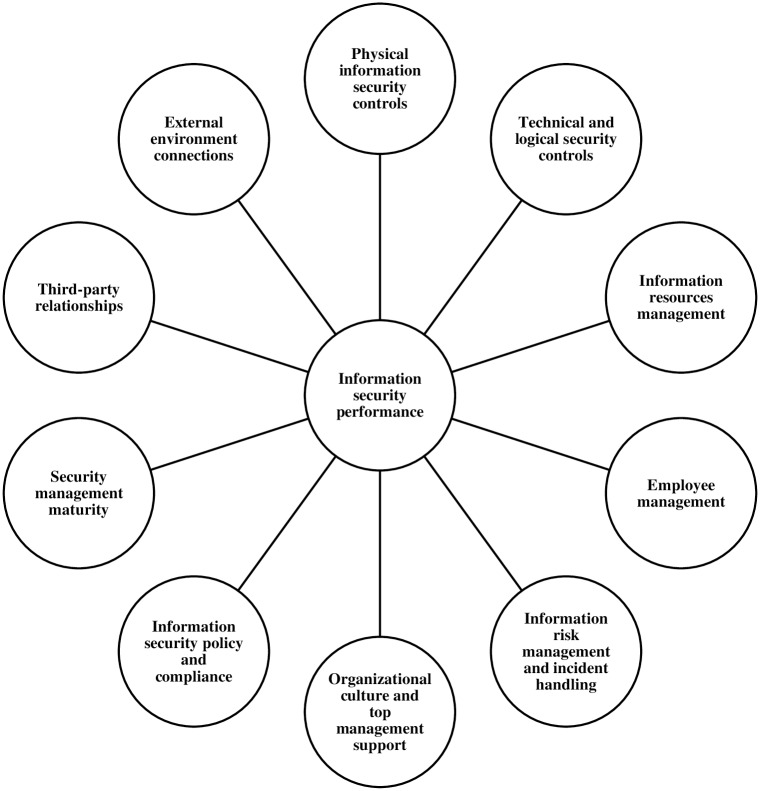
Simplified representation of information security performance system.

The first step in compiling the model’s content involved the identification of CSFs influencing the object of measurement, i.e. information security performance. These factors were defined by considering the conditions or measures discussed in Section 2.2. The ISP 10×10M foresees that organizations must simultaneously develop their information security in different areas (as shown in Fig 1) related to operational, tactical and strategic activities. These ten areas constitute CSFs or, in other words, dependent variables that have the greatest impact on information security in organizational environments.

During the next step of this phase, each factor was further defined by KPIs or, in other words, independent variables used to assess the performance of individual areas, which were developed on the basis of findings obtained from the descriptive analysis of standards presented in S1 Table. Indicators comprise organizational measures and security controls that need to be implemented in order to guarantee maximum performance of the aforementioned information security areas. Each factor is defined by ten independent variables. This led to the creation of the 10 by 10 information security performance measurement model—ISP 10×10M, composed of 10 CSFs and 100 (10×10) KPIs. The content of the model is presented in Table 1.

**Table 1:** CSFs and KPIs constituting the model.

Critical success factors	Key performance indicators	
**1. Physical information security controls**	1.a	Fire, voltage and flood protection of buildings and premises
	1.b	Adequate installation and management of communication and power network
	1.c	Support systems for critical services—power supply, cooling, communication
	1.d	Control of third-party access to buildings and premises
	1.e	Control of employee access to buildings and premises
	1.f	Adequate installation and physical protection of hardware
	1.g	Regular maintenance of hardware
	1.h	Protection of ICT located outside organizations’ premises (MDM systems)
	1.i	Adequate building architecture and security plans in place—defined security areas
	1.j	Protection of buildings and premises against break-ins and wiretapping
**2. Technical and logical security controls**	2.a	Malware protection
	2.b	Logical security of programs, systems and databases—identification/authorization
	2.c	Technical protection of local networks (LAN) and network devices
	2.d	Technical measures aimed at protecting information during their storage
	2.e	Technical measures aimed at protecting communications and information during transfer
	2.f	Access control—log management, activity monitoring
	2.g	Adequate system capabilities and capacities for information processing—system reliability
	2.h	Standardization of workstations
	2.i	Change management—analyses of impacts that technology changes have on existing systems
	2.j	Regular (automated) security updates of software and systems
**3. Information resources management**	3.a	Security categorization of information
	3.b	Defined administration and other responsibilities related to information management
	3.c	User guidelines for handling information
	3.d	Implementation of the “need-to-know” principle
	3.e	Control over the exercise of administrator and system rights
	3.f	Definition and protection of organization’s intellectual property
	3.g	Definition and protection of personal data
	3.h	Provision of data processing traceability—audit trails
	3.i	Adequate deletion of data, destruction of equipment and physical documentation
	3.j	Information archiving and back-ups
**4. Employee management**	4.a	Raising employees’ awareness regarding information risks and policies
	4.b	Defined responsibilities related to the use of confidential systems and data
	4.c	Defined disciplinary proceedings, sanctions and infringement proceedings
	4.d	User rights management throughout employment—before, during, after employment
	4.e	Security vetting of employees
	4.f	Employee agreements and declarations concerning the protection of confidentiality
	4.g	Provision of technical and consultative support to employees
	4.h	Defined remote access and teleworking procedures
	4.i	Protection of employee rights during information security control procedures—protection of privacy
	4.j	Professional training of security and technical personnel
**5. Information risk management and incident handling**	5.a	Business continuity plan and policy
	5.b	Automated early warning systems—IDS, IPS, SIEM
	5.c	Defined procedures for reporting and handling detected irregularities
	5.d	Crisis management—plans for responding to critical security risks
	5.e	An alternative location (i.e. hot spot) for the most important parts of information systems
	5.f	Incident monitoring, recording and analysis—experiential learning
	5.g	Forensic procedures and evidence gathering for incident investigations
	5.h	Information risk management—analysis and evaluation
	5.i	Analyses of former information incidents’ impacts on business operation—damage assessment
	5.j	Assessment of existing security controls’ efficiency—performance measurement
**6. Organizational culture and top management support**	6.a	Ethical, socially responsible and transparent security management
	6.b	Pursuing the principle of efficiency in information security—economy/cost optimization
	6.c	Good relations and constructive debates regarding security controls between organizational departments
	6.d	Inclusion of information security in the planning of organizational projects and changes
	6.e	Leadership familiarity with security needs—open communication channels
	6.f	Users’ general satisfaction and confidence with respect to information security
	6.g	Organizations’ innovativeness, excellence and continuous development in the field of IT
	6.h	Adequate staffing and financial support to information security
	6.i	Clearly defined organizational hierarchy and job classification
	6.j	Leaderships’ involvement in information security planning
**7. Information security policy and compliance**	7.a	Adoption of a formal information security policy
	7.b	Policy’s breakdown into sub-areas and orderly documentation
	7.c	Monitoring the respect of policies among users during their everyday work
	7.d	Compliance with international standards and recommendations
	7.e	Continuous development and upgrading of information security—control of risks and conformity
	7.f	Regular management reviews and internal audits
	7.g	Compliance with relevant legislation
	7.h	Fulfillment of contractual security obligations
	7.i	Use of licensed products and services
	7.j	Analysis of examples of information security best practices—benchmarking
**8. Security management maturity**	8.a	Strategic and long-term planning of information security—proactive approach
	8.b	Development of information security as a business function or special department/service
	8.c	Adequate personnel structure—recruitment of qualified staff
	8.d	Formal authority of security personnel—ability of decision-making
	8.e	Division between system-related and security tasks—separation between IT and security division
	8.f	Cooperation with other organizational authorities in information security planning
	8.g	Regular vertical and horizontal security meetings
	8.h	Team decision-making regarding management of critical security risks
	8.i	Management of employees’ security culture and motivational activities
	8.j	Legitimacy of information security—compliance with user requirements
**9. Third-party relationships**	9.a	Formalized contractual relationships with partners and suppliers regarding information security
	9.b	Defined security responsibilities with respect to customers
	9.c	Involvement of third parties in the implementation of information security measures
	9.d	Good customer relations—building trust and reputation/organizations’ goodwill
	9.e	Testing ICT before acquisition—defined acceptability criteria and quality
	9.f	Security vetting of business partners and suppliers
	9.g	Defined and regulated security of e-business
	9.h	Adequate technical protection of inter—organizational information systems
	9.i	Formalized contractual relationships for the processing and exchange of personal data
	9.j	Liability insurance covering information security events and incidents
**10. External environment connections**	10.a	Flexibility of organizations—adapting to changes in the sector and the environment
	10.b	Successful management of competitive and external pressures
	10.c	Cooperation with other sectoral organizations—inter-organizational strategic ties
	10.d	Participation in economic and business associations, societies and groups
	10.e	Cooperation with competent authorities when dealing with information incidents
	10.f	Cooperation with security consultant groups and external audits of information security
	10.g	Active participation in foreign environments—international cooperation for knowledge sharing
	10.h	Defined rules governing communication with the public and competitive organizations
	10.i	Monitoring technological developments and implementing innovations regularly
	10.j	Monitoring and analyzing security trends—development of threats and vulnerabilities

In its initial stages, the model contained more variables and areas of measurement, which is why it was discussed with three practitioners before it was further developed. A faculty member from the information security field, an owner of a small Slovene company and a personal data inspector reviewed the model and presented their recommendations and opinions. After considering their comments, the number of areas and criteria were reduced to the form presented in the 10 by 10 model

### Sample and Data Collection

While compiling the criteria we deliberated different possibilities for devising an assessment method that would be as simple as possible on one hand and reliable on the other. It quickly became clear that this cannot be achieved without weighting individual criteria. In truth, factors and indicators cannot be considered equally important due to their diverging impacts on the final state-of-play in information security. Naturally, their actual impact depends on the organizational context. However, they need to be generalized in order to be applicable to all organizations, which is why the extent to which individual measures (indicators) are important for establishing good practices had to be identified. In order to guarantee objective values of weighting coefficients, the model and its content were evaluated by practitioners, who are dealing with information security on a daily basis, are familiar with organizations’ needs and difficulties, and are thus able to provide relevant assessments. The first step of the second phase of model’s development was conducted through an empirical analysis. Because a high rate of unresponsiveness is a relatively normal and frequent phenomenon in the field of security research [12, 50, 58], we decided to test the model by adopting a different, i.e. interactive approach.

Data used to weight key performance indicators were collected during a single group presentation of the model and its evaluation, which took place in the form of an interactive lecture. The model was tested in March 2014 during a lecture entitled “Analyzing information security performance through an interactive approach”, which was given at the Risk 2014 conference. Prior to conducting the study, the research proposal was approved by the Senate of the Faculty of Criminal Justice and Security, University of Maribor. The Risk conference, where the data gathering process took place, is an international conference dedicated to the security of new technologies and trending services, where experts from IT and security professions and government organizations meet and discuss emerging issues. All conference participants were invited to partake in the study via the conference program. At the beginning of the section, they were informed that the participation is voluntary and anonymous, and that the gathered data would only be published in aggregated form. They were given an explanation about the purpose of the study and informed that the study was only intended for information security professionals and/or people whose work duties and responsibilities include information security issues. Out of more than 60 people who attended the process, 43 participated in the study. This (43) was the number of remotes connected to the Turning point polling software [59] and therefore available for data collection. Since those who decided to participate identified themselves as a target group and willingly and fully voluntarily attended our section, it is reasonable to assume that the sample consisted fully of information security experts, who consented to the participation in our study. The demographic description of the sample is provided in Table 2, which shows that participants consisted of qualified IT experts mostly representing computer, telecommunication and financial organizations.

**Table 2:** Sample demography.

Demography	Area	Percentage
**Qualification**	Primary education or lower	2.6%
	Secondary education	13.2%
	National vocational qualification	7.9%
	Higher-education or university qualification	60.5%
	Masters or doctoral qualification	15.8%
**Sector**	Public sector	35.9%
	Private sector	64.1%
**Organization’s business activity**	Scientific activity: education, research	7.1%
	Computer science, telecommunications	28.6%
	Security consultancy or auditing	4.8%
	Financial institution, insurance undertaking	16.7%
	Healthcare sector	2.4%
	Construction sector	2.4%
	Transport sector	4.8%
	Electronics and electricity sector	19%
	Other	14.3%
**Department/profession**	IT	71.4%
	Security	9.5%
	Finance, accounting	2.4%
	Sales, marketing	4.8%
	Other	11.9%

Data were collected by using the Turning Point v.5 [59] interactive hardware (remote controls) and software (turning point survey embedded into a PowerPoint presentation). The data collection process began by first presenting the purpose and content of the model, and the goal of the lecture. This was followed by an interactive explanation of each individual factor and indicator, the importance of which was simultaneously evaluated by participants using the aforementioned remotes connected to the software by assigning (i.e. clicking for answer) a value from 1 to 5 on the Likert scale. The software recorded and stored their answers. Participants were asked the following question: “What is the importance of individual criteria for an efficient implementation of information security?” When assigning the level of importance, value 1 meant that an indicator was less important, while value 5 meant that an indicator was of critical importance and that it must necessarily be implemented by every organization.

During a one-hour presentation, participants evaluated a total of 114 variables. This enabled us to collect data regarding the level of importance of individual variables or, in other words, establish how much attention organizations should pay to individual areas and measures in order to guarantee the highest level of information security performance. The obtained evaluations enabled the implementation of the second step in this phase, which was aimed at verifying the fulfillment of formal statistical conditions and calculate indicators’ weights.

## Data Analysis and Results

The analysis and processing of collected data were conducted by using the SPSS v.22 statistical software [60]. A preliminary review of data identified the occurrence of missing data (missing values). When reviewing the samples by applying a pattern analysis and Little’s MCAR test (Chi-Square = 47,463, DF = 3880, Sig. = 1), it was identified that there were no particular patterns and that missing values were distributed randomly. Therefore, the observed data could be used for further statistical analysis.

The reliability of the research construct was verified by analyzing the inter-connectedness and inter-dependence of criteria. Model’s internal consistency is demonstrated by analyzing the Cronbach’s alpha coefficient. In social science research, the value of such a coefficient should be higher than 0.8 in order to demonstrate a strong reliability of the research construct. In this case, Cronbach’s alpha for observed data amounts to 0.917 for 110 measurement items, which indicates that the research construct is reliable and there is a strong correlation between criteria [61].

The reliability of responses and the avoidance of the error of the first kind were also influenced by the actual data collection procedure, which was anonymous, voluntary, pressureless and unbiased, and provided for all respondents to listen to the same explanation of the model and its criteria. The first step in the second phase of creating the model thus confirmed the validity and reliability of its content set during the first phase.

Apart from reliability, the research construct was also verified with respect to its validity in order to establish whether the content of the model is actually measuring the features that were predetermined as the object of measurement. Firstly, we verified the normal distribution. The z-values of skewness fell below 2, while all kurtosis fell within the category of +/- 3, which indicated that the data in our study were close to the univariate normality and were thus eligible for the performed statistical analyses that followed [62]. The values shown in Tables 3 and 4 (0.00–1.67 for skewness and 0.02–2.8 for kurtosis) can be considered and accepted as normal. Two responses (one for indicator 1.b and one for indicator 3.j) were excluded as outliers for the purpose of achieving data normality. Furthermore, we tested the means of all variables with a one-sample t-test (test value of 2.5). The results show that the calculated average values exceed the proposed estimation and are statistically significant (p-value < 0.05), which applies to all variables respectively. This also indicates that all measurement items relate to the object of measurement. Additionally, we also applied one-sample Wilcoxon signed rank tests to determine the extent of importance of the criteria for information security performance. Median values of all variables were verified in order to determine their relevance. It was established that the median value of factors and areas in observed data never falls below 3.4, which significantly exceeds the median rate and proves that (according to participating experts) all criteria have a strong impact on information security. Standard deviations (SD) of data prove that responses were not excessively dispersed and that they were distributed properly around the mean value, while values of the standard errors of the mean (SE), if confidence intervals of the means are calculated (i.e. 1.96×SE +/- $\bar{x}$), confirm that the ranking of variables, which took place later on, is generally fair with respect to the computed mean.

**Table 3:** CSFs categorization.

CSF	n	$\bar{x}$	SME	SD	Skew.	Kurt.	Median (p > 0,05)	FW
5	42	4.24	.140	.906	-1.331	2.360	4.5	1.4
3	40	4.23	.127	.800	-.754	-.022	4.5	1.3
4	40	4.10	.142	.900	-.648	-.462	4.4	1.2
1	39	4.05	.160	.999	-.942	.725	4.4	1.1
2	42	4.00	.128	.826	.000	-1.538	4.4	1.05
6	38	3.71	.136	.835	.017	-.638	3.9	0.95
8	42	3.57	.128	.831	.164	-.521	3.9	0.9
7	41	3.56	.152	.976	-.519	.738	3.9	0.8
10	40	3.43	.160	1.010	-.178	-.424	3.9	0.7
9	42	3.38	.167	1.081	-.103	-.851	3.5	0.6

**Table 4:** KPI categorization.

CSF	KPI	n	$\bar{x}$	SE	SD	Skew.	Kurt.	Median (p > 0,05)	IW	W
1. Physical information security controls (FW = 1.1)	1.b	40	4.53	.101	.640	-1.024	.041	4.9	0.28	0.308
	1.c	39	4.49	.096	.601	-.714	-.393	4.5	0.26	0.286
	1.a	40	4.30	.140	.883	-.880	-.480	4.5	0.24	0.264
	1.g	41	4,.12	.145	.927	-1.241	2.039	4.4	0.22	0.242
	1.d	41	4.10	.167	1.068	-1.109	.592	4.5	0.21	0.231
	1.f	41	4.00	.171	1.095	-1.320	1.360	4.4	0.19	0.209
	1.h	42	3.95	.132	.854	.094	-1.639	4	0.18	0.198
	1.e	41	3.80	.165	1.054	-.397	-1.027	4	0.16	0.176
	1.j	41	3.29	.182	1.167	-.213	-.660	3.5	0.14	0.154
	1.i	39	3.28	.156	.972	-.249	-.526	3.5	0.12	0.132
2. Technical and logical security controls (FW = 1.05)	2.c	40	4.50	.101	.641	-.924	-.136	4.9	0.28	0.294
	2.b	40	4.50	.107	.679	-1.033	-.086	4.9	0.26	0.273
	2.a	41	4.39	.115	.737	-.780	-.710	4.5	0.24	0.252
	2.j	37	4.30	.149	.909	-1.115	.334	4.5	0.22	0.231
	2.f	40	4.25	.117	.742	-.445	-1.028	4.5	0.21	0.2205
	2.e	38	3.97	.153	.944	-.759	.843	4.4	0.19	0.1995
	2.d	39	3.82	.151	.942	-.816	.921	4	0.18	0.189
	2.g	41	3.59	.135	.865	-.033	-.569	3.9	0.16	0.168
	2.h	39	3.59	.179	1.117	-.297	-.790	3.9	0.14	0.147
	2.i	40	3.33	.158	.997	-.386	.035	3.5	0.12	0.126
3. Information resources management (FW = 1.3)	3.j	38	4.63	.088	.541	-1.100	.241	4.9	0.28	0.364
	3.g	39	4.18	.176	1.097	-1.131	.423	4.9	0.26	0.338
	3.e	38	4.13	.197	1.212	-1.131	-.040	4.9	0.24	0.312
	3.b	39	4.08	.124	.774	-.495	-.099	4.4	0.22	0.286
	3.a	39	4.00	.168	1.051	-1.289	1.743	4.9	0.21	0.273
	3.c	38	3.84	.158	.973	-.781	.664	4	0.19	0.247
	3.h	38	3.76	.162	.998	-.869	.511	4	0.18	0.234
	3.i	39	3.54	.179	1.120	-.160	-.860	3.9	0.16	0.208
	3.d	37	3.49	.148	.901	-.439	.497	3.9	0.14	0.182
	3.f	38	3.45	.159	.978	-.302	-.178	3.9	0.12	0.156
4. Employee management (FW = 1.2)	4.j	38	4.26	.163	1.005	-1.579	2.333	4.5	0.28	0.336
	4.a	40	4.18	.133	.844	-.350	-1.517	4.4	0.26	0.312
	4.h	39	4.03	.125	.778	-.399	-.250	4.4	0.24	0.288
	4.b	40	4.00	.139	.877	-.480	-.516	4	0.22	0.264
	4.i	36	3.86	.188	1.125	-.732	-.297	3.9	0.21	0.252
	4.d	37	3.84	.167	1.014	-.840	.480	4	0.19	0.228
	4.c	34	3.65	.152	.884	-.057	-.640	3.9	0.18	0.216
	4.f	39	3.56	.151	.940	-.395	,244	3.9	0.16	0.192
	4.g	39	3.51	.146	.914	-.585	.380	3.9	0.14	0.168
	4.e	36	2.94	.169	1.013	-.059	-.214	3.4	0.12	0.144
5. Information risk management and incident handling (FW = 1.4)	5.e	38	4.13	.142	.875	-.778	-.027	4.4	0.28	0.392
	5.a	38	4.08	.166	1.024	-.963	.592	4.4	0.26	0.364
	5.b	38	3.95	.141	.868	-.418	-.490	4.4	0.24	0.336
	5.c	36	3.83	.116	.697	.238	-.843	4	0.22	0.30
	5.d	39	3.82	.164	1.023	-.710	.175	4	0.21	0.294
	5.f	39	3.69	.128	.800	-.023	-.441	3.9	0.19	0.266
	5.g	38	3.58	.191	1.177	-.672	-.120	3.9	0.18	0.252
	5.h	34	3.50	.170	.992	-.395	-.066	3.9	0.16	0.224
	5.i	36	3.28	.167	1.003	-.423	-.021	3.5	0.14	0.196
	5.j	37	3.24	.183	1.116	-.639	-.209	3.5	0.12	0.168
6. Organizational culture and top management support (FW = 0.95)	6.h	38	4.08	.122	.749	-.131	-1.156	4.4	0.28	0.266
	6.c	35	4.06	.147	.873	-.678	-.088	4.4	0.26	0.247
	6.d	38	4.00	.156	.959	-1.166	1.634	4.4	0.24	0.228
	6.j	37	3.84	.148	.898	-.151	-.896	4	0.22	0.209
	6.e	41	3.78	.146	.936	-.305	-.722	4	0.21	0.1995
	6.i	38	3.68	.173	1.068	-1.002	.607	4	0.19	0.1805
	6.f	36	3.64	.179	1.073	-.677	.329	4	0.18	0.171
	6.g	39	3.51	.176	1.097	-.664	.279	3.9	0.16	0.152
	6.b	30	3.43	.177	.971	-1.008	1.068	3.9	0.14	0.133
	6.a	37	3.43	.196	1.191	-.091	-.781	3.9	0.12	0.114
7. Information security policy and compliance (FW = 0.8)	7.g	36	4.03	.167	1.000	-.967	.882	4.4	0.28	0.224
	7.a	38	3.89	.176	1.085	-1.254	1.265	4.4	0.26	0,.208
	7.h	38	3.84	.167	1.027	-.299	-1.120	4	0.24	0.192
	7.i	38	3.84	.218	1.346	-1.030	-.101	4.4	0.22	0.176
	7.e	38	3.79	.152	.935	-.812	.981	4	0.21	0.168
	7.c	35	3.66	.136	.802	-.367	-.087	3.9	0.19	0.152
	7.d	37	3.65	.130	.789	-.343	-.077	3.9	0.18	0.144
	7.f	37	3.41	.147	.896	-.189	.446	3.5	0.16	0.128
	7.b	33	3.36	.173	.994	-.206	-.248	3.5	0.14	0.112
	7.j	37	3.35	.191	1.160	-.408	-.457	3.9	0.12	0.096
8. Security management maturity (FW = 0.9)	8.i	37	3.86	.146	.887	-.481	-.314	4	0.28	0.252
	8.c	39	3.85	.135	.844	-.247	-.531	4	0.26	0.234
	8.b	37	3.76	.166	1.011	-.330	-.928	4	0.24	0.216
	8.d	39	3.69	.202	1.260	-.877	-.027	4	0.22	0.198
	8.a	37	3.65	.170	1.033	-.503	-.174	3.9	0.21	0.189
	8.j	38	3.53	.195	1.202	-.558	-.402	3.9	0.19	0.171
	8.h	37	3.51	.172	1.044	-.270	-.455	3.9	0.18	0.162
	8.f	35	3.46	.171	1.010	-.330	-.285	3.9	0.16	0.144
	8.e	36	3.25	.166	.996	-.539	.628	3.5	0.14	0.126
	8.g	37	3.14	.141	.855	.575	.016	3.4	0.12	0.108
9. Third-party relationships (FW = 0.6)	9.g	39	4.05	.176	1.099	-1.234	1.256	4.4	0.28	0.168
	9.a	41	4.00	.185	1.183	-1.333	1.148	4.4	0.26	0.156
	9.d	38	3.95	.164	1.012	-.881	.578	4.4	0.24	0.144
	9.b	40	3.88	.176	1.114	-1.031	.605	4.4	0.22	0.132
	9.e	39	3.82	.190	1.189	-1.218	1.055	4.4	0.21	0.126
	9.j	35	3.80	.187	1.106	-.963	.644	4	0.19	0.114
	9.i	33	3.76	.200	1.146	-.948	.350	4	0.18	0.108
	9.h	36	3.47	.189	1.134	-.550	.034	3.9	0.16	0.096
	9.f	37	3.43	.211	1.281	-.637	-.424	3.9	0.14	0.084
	9.c	40	3.40	.142	.900	-.684	1.138	3.5	0.12	0.072
10. External environment connections (FW = 0.7)	10.i	38	4.13	.169	1.044	-1.629	2.811	4.5	0.28	0.196
	10.j	38	4.13	.189	1.166	-1.671	2.282	4.5	0.26	0.182
	10.a	39	3.67	.174	1.084	-.455	-.511	4	0.24	0.168
	10.e	36	3.53	.180	1.082	-.506	.002	3.9	0.22	0.154
	10.f	38	3.50	.202	1.247	-.618	-.385	3.9	0.21	0.147
	10.h	38	3.42	.167	1.030	-.638	.698	3.9	0.19	0.133
	10.b	40	3.38	.205	1.295	-.381	-.935	3.9	0.18	0.126
	10.c	38	3.13	.197	1.212	-.265	-.636	3.5	0.16	0.112
	10.g	39	3.10	.207	1.294	.108	-1.045	3.5	0.14	0.098
	10.d	37	2.92	.175	1.064	-.270	-.814	3.4	0.12	0.084

The evaluation of the model’s reliability and validity was followed by the second step of the phase dedicated to assessing the importance of variables and their weighting. For this purpose, descriptive statistics, i.e. mean values ($\bar{x}$), standard deviations (SD), standard errors of the mean (SE), were calculated. This enabled a categorization on the scale from the most to the least important variable (Tables 3 and 4), where the categorization of variables was conducted by considering the M and SD. Factors were weighted with values (FW) from 0.6 to 1.4, where the most important CSFs according to experts’ opinion were attributed the highest value, while the lowest value was assigned to the least important CSFs. A similar approach was adopted for weighting KPIs within each individual CSF with values (IW) from 0.12 to 0.28. Weights remained constant through the subsequent computation process—all KPIs within individual CSFs that were evaluated as the most or the least important by experts were assigned a weight amounting to 0.28 or 0.12 respectively. All weights were equally increased, but no CSFs or KPIs within individual CSFs were allocated the same weight. Total weights (W), which are unique to each KPI, were calculated by multiplying a KPI’s constant weight with the constant weight of a CSF that includes a particular KPI (W = FW x WI). Descriptive statistics and weights attributed to CSFs and KPIs are presented in the below Tables 3 and 4, where KPIs within individual CSFs are categorized from the most to the least important (second column).

The weight values for CSFs and KPIs were determined and selected with a view to devise a simple model. Organizations use a questionnaire and a 5-level scale (1–5) to evaluate the extent to which they implemented and developed individual measures in practice (100 indicators in total). In order for the final results to be as clear and understandable as possible, the maximum limit organizations can reach (when developing all measures) is 100, while the lowest amounts to 20. Since weights must increase evenly and each indicator requires a unique total weight, the selected method (factor’s weight x indicator’s weight = total weight) represents the only possible way for achieving such maximum and minimum limits (sum (total weight _1–100_ × 5) = 100; sum (total weight _1–100_ × 1) = 20) in order to avoid the normalization of final results. Normalization of results is an alternative approach, but the process of calculating comprehensible final results would become more complex and intricate.

An example of computing W: KPI 1.a belongs to CSF 1 (Physical information security controls), which ranks fourth (out of ten) on the scale of CSF’s importance and is thus assigned the weight of 1.1. KPI 1.a. (Fire, voltage and flood protection of buildings and premises) ranks third on the scale within this CSF and is assigned the weight of 0.24. The total weight of this KPI thus amounts to 1.1 × 0.24 = 0.264.

**Fig 2:**
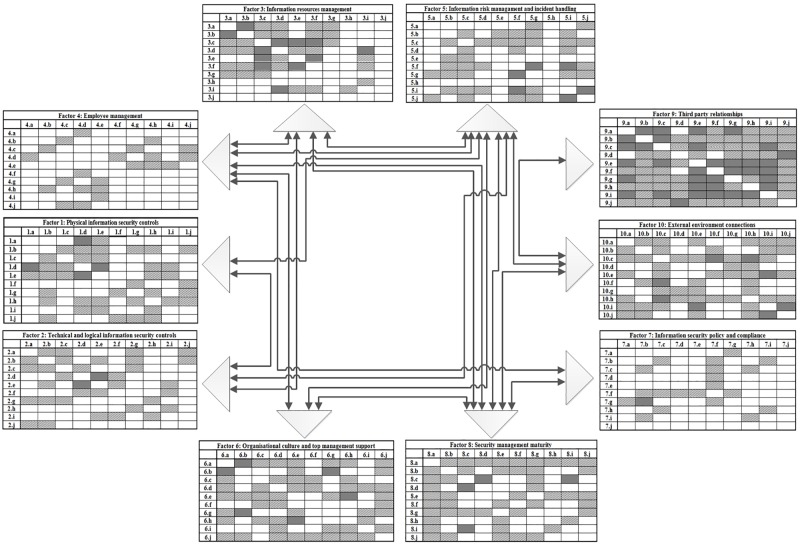
Model of variable correlations.

Apart from determining weights, the statistical analysis also served as a basis for establishing correlations between CSFs and individual KPIs within CSFs. The procedure was conducted by calculating the Pearson correlation coefficients between CSFs and between KPIs included in individual CSFs. The analysis of correlations seen in Fig 2 shows, for instance, that CSF 7 (Information security policy and compliance) is most strongly correlated with CSF 4 (Employee management) and CSF 8 (Security management maturity). If an organization had to improve the formal area of information security and compliance, it would have to enable the development of measures in the two aforementioned areas. Such correlations are reasonable and logical, since the competences of security management influence the quality of security policy definition and the clarity of set rules and regulations, while the extent to which these rules and regulations are respected in practice depends on employees. Apart from these correlations, there are also other significantly correlated areas. Fig 2 demonstrates the links between other areas and variables. The third, fourth, fifth and eighth factor are the most influential, while the maximum internal correlations between individual measures are observed in the third, ninth and tenth factor. These findings may be useful for further analysis of the situation in a specific organizational environment in terms of identifying the best possible solutions and recommendations for the management.

In the third phase, the obtained scale of criteria and their weights were used to establish the method for calculating performance and critical limits. The ISP 10×10M presumes that organizations ought to evaluate the extent to which they meet individual criteria by filling in a questionnaire in the form of a checklist. This is done by using the Likert scale from 1 to 5, where 1 means that a measure is not adopted and 5 means that a measure is fully implemented in practice. The final result of information security performance is then obtained by calculating the sum of values given to all indicators and multiplying these with organizations’ evaluations. If, for instance, an organization estimates that KPI 1.a deserves the average mark (3) on a scale from 1 to 5, this mark should then be multiplied with the weight assigned to this indicator, as follows: 0.264 × 3 = 0.792.

The highest possible result that organizations can achieve (if they believe that all measures are implemented in practice) amounts to 100, while the lowest result equals 20 (if no measures are developed).

**Fig 3:**
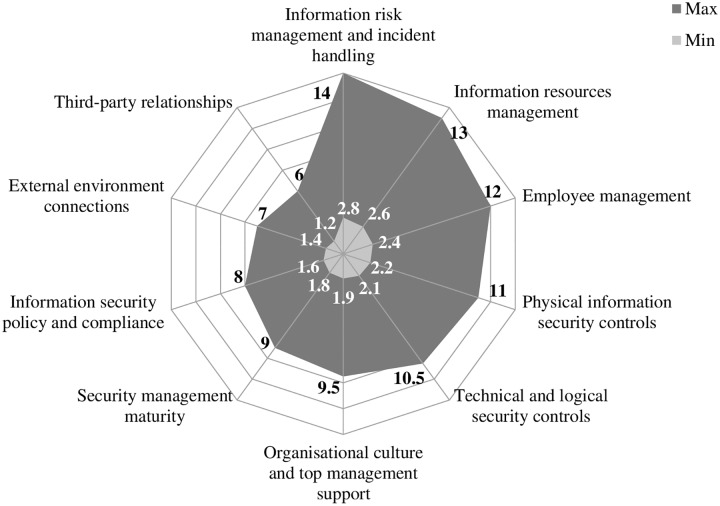
Examples of the best and worst information security performance results.

By analyzing results and establishing the importance of the model’s individual elements, the optimum security situation in an organization can also be presented graphically. The polar chart in Fig 3 presents three information security situations or the number of points an organization can obtain in an individual area by investing minimum and maximum efforts. Fig 3 also shows which areas have the strongest and the weakest impact on information security performance. The ISP 10×10M presumes that a holistic information security system can only be achieved by providing a high level of performance in ten different fields. However, absolute or 100% security does not exist and cannot actually be achieved in practice, which is why organizations are only partially successful, even though they invest maximum efforts. The white area denotes the absolute information security performance composed of ten factors, which is only achievable in theory; the dark area shows the optimum level of information security performance achieved when organizations efficiently manage all areas; while the grey area presents a situation, in which organizations are not developing any of the factors.

The result achieved through the use of the ISP 10×10M enables the categorization of organization into one of the information security levels. We established three principal levels, i.e. basic, intermediate and advanced level. Each principal level is further divided into two sub-levels, which makes up a total of six sub-levels.

The aforementioned individual levels and sub-levels were further used to categorize KPIs of the model and for creation of its final form, which was done in a way that all three levels contain measures foreseen by all ten CSFs. This allows for a clear and accurate presentation of measures that should be developed by organizations if they wish to meet the requirements of individual levels or advance to a higher level. In order to provide a clearer picture, the model, together with its levels, criteria and critical values, is presented in the Table 5. Titles of individual KPIs were simplified, since their detailed presentation is available in Table 1.

**Table 5:** ISP 10×10M layout.

F	Basic	W	Intermediate	W	Advanced	W
	***Level 1***		***Level 3***		***Level 5***	
**5**	Hot-site Business continuity Warning and detection	0.392 0.364 0.336	Reporting and recording Crisis management Incident analysis Digital and network forensic	0.308 0.294 0.266 0.252	Risk management Incident impact analyses Performance assessment	0.224 0.196 0.168
**3**	Back-ups Personal data protection User rights control	0.364 0.338 0.312	Defined ownership Data classification User guidelines Audit trails	0.286 0.273 0.247 0.234	Safe data destruction “Need-to-know” policy Intellectual property protection	0.208 0.182 0.156
**4**	Professional training Information security awareness Remote access	0.336 0.312 0.288	Defined responsibilities Protection of employee rights User rights status management Disciplinary policy	0.264 0.252 0.228 0.216	Employee declarations Technical support Security vetting	0.192 0.168 0.144
**1**	Managing information system infrastructure Support systems Disaster protection	0.308 0.286 0.264	Regular hardware maintenance Control of third-party access Physical protection of hardware Protection of mobile ICT	0.242 0.231 0.209 0.198	Access control Anti-theft system Defined security zones	0.176 0.154 0.132
**2**	Network protection Identification/authorization Malware protection	0.294 0.273 0.252	Automatic security updates Access control to systems, databases Protection of communications Protection of stored information	0.231 0.221 0.200 0.189	System capabilities and capacities Workstation standardization Configuration management	0.168 0.147 0.126
	***Level 2***		***Level 4***		***Level 6***	
**6**	Adequate HR and financial support Good employee relations Inclusion in projects	0.266 0.247 0.228	Management’s involvement and support Open communication channels Clearly defined hierarchy Users’ general satisfaction	0.209 0.200 0.181 0.171	Innovativeness, excellence Economy/cost optimization Goodwill, transparency	0.152 0.133 0.114
**8**	Security culture Qualified staff Information security department	0.252 0.234 0.216	Designated and authoritative management Strategic planning Legitimacy Team decision making	0.198 0.189 0.171 0.162	Inter-sectoral cooperation Division of tasks Regular security meetings	0.144 0.126 0.108
**7**	Regulatory compliance Adopted formal policy Contractual obligations	0.224 0.208 0.192	Use of licensed products Continuous development, upgrading Monitoring the respect of policies Compliance with standards	0.176 0.168 0.152 0.144	Continuous audits Analysis of best practices Policy’s breakdown	0.128 0.112 0.096
**10**	Monitoring ICT development Security trends Organizations’ flexibility	0.196 0.182 0.168	Cooperation with authorities External audits Public communication governance Benchmarking and competition control	0.154 0.147 0.133 0.126	Strategic inter-org. collaboration International cooperation—knowledge sharing Participation in security specialized associations	0.112 0.098 0.084
**9**	E-business security Partner relationships Good relations, goodwill	0.168 0.156 0.144	Defined responsibilities to customers Testing ICT before acquisition Liability insurance Regulated processing of personal data	0.132 0.126 0.114 0.108	Inter-organizational security Vetting of partners Involvement of third parties	0.096 0.084 0.072

Min: 20 points

Max: 100 points

**Basic score: 20–40**
Sub-level 1 score: 20–30 Sub-level 2 score: 31–40

**Intermediate score: 41–80**
Sub-level 1 score: 41–60 Sub-level 2 score: 61–80

**Advanced score: 81–100**
Sub-level 1 score: 81–90 Sub-level 2 score: 91–100

Table 5 clearly shows that the basic level comprises those KPIs within individual CSFs that were evaluated as the most important by experts, while the advanced level includes those KPIs that were evaluated as less significant, at least at the initial stages of system establishment. Those measures are more specific and complex, and normally in the domain of the most developed and responsible organizations in terms of security. Both basic and advanced levels contain three measures from each CSFs, while the intermediate level includes four such measures. Each of the three levels is further composed of the first and second sub-level. The first sub-level contains the top five most important CSFs according to experts’ evaluation, while the second sub-level contains the remaining five CSFs. Based on the selected categorization, the first sub-level of each level presupposes that priority should be given to the development of operational aspects related to specific repressive and restrictive measures (technical, logical and physical controls) in the field of information security. On the other hand, the second sub-level includes CSFs and KPIs that are of distinctly strategic and socio-preventive nature, since they refer to long-term planning and the adaptation of information security to organizational, formal, user and environmental requirements.

**Fig 4:**
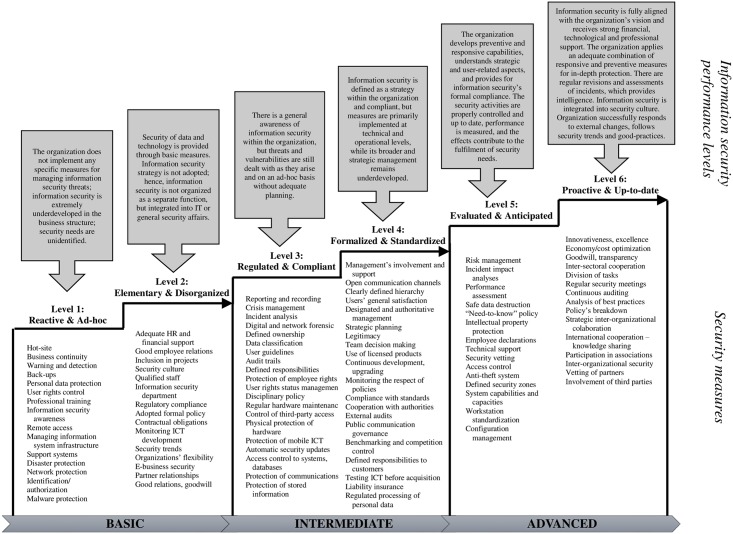
Information security performance levels according to the ISP 10×10M.

The visual representation of such a model structure is provided in Fig 4. In each of the “staircases”, where an organization finds itself when performing an evaluation, a performance level is described and backed-up with measures that need to be considered in each of the ten information security areas.

Such a set-up of the model allows organizations to quickly and easily identify those factors or areas of information security that need to be addressed and specific criteria that need to be met in order to satisfy the needs established by the level and sub-levels, in which they were placed. Organizations can thus obtain recommendations regarding the necessary improvements and measures.

## Discussion

This paper presents the research that served as a basis for building the ISP 10×10M, which in many ways complements and upgrades existing efforts in the field of information security. In terms of its content, the model and its formation comply with the findings of other empirical studies. For instance, many experts emphasize that information security is efficient when organizations simultaneously develop their preventive and responsive capabilities [13, 28, 47]. The research presented herein complements that, since information risk management ranks first, i.e. the most important CSF of the model, while its KPIs clearly define the aforementioned combinations of measures. In this context, the existence of an alternative location, business continuity and early warning systems were identified as the most important indicators. These are followed by other significant indicators, which include information back up, protection of personal data, control over user rights, professional training, raising employees’ awareness and secure remote access procedures. These measures are related to the management of information sources and users. These three factors (information risk management, management of information sources and employee management) are followed by technical, logical and physical controls, which generally represent one of the first steps organizations (should) take when planning and establishing information security in practice [63]. Research studies conducted by Ernst&Young, BIS and PWC also confirm our conclusion; they indicate that business continuity, recovery procedures and measures for preventing outside attacks represent those areas of information security that require priority treatment [20, 23]. Reports from PWC and Ponemon Institute conclude that the most efficient measures from the point of view of cost-benefit analyses include technical measures or intelligent threat detection systems (SIEM, IPS, IDS, UTM, NGFW, etc.), while protection against malware, encryption, network protection and logical controls are of particular importance as well. Apart from detection systems, other profitable and advisable measures include the recruitment of appropriate professional staff responsible for information security and activities falling in the scope of employee management, such as the management of user rights and control of employee access to systems [21, 64].

Experts participating in the research categorized the formal conditions or compliance of information security as a less important (albeit not insignificant) area, compared to the aforementioned factors. They believe that the fulfillment of formal conditions does not have as strong an impact as, for instance, technical protection and user management. The same findings were presented by Deloitte [65], which states that compliance with regulatory requirements does not have a significant impact in terms of information security performance and that organizations no longer consider it a priority. Standardization procedures are advisable mainly for larger and more complex organizations, while smaller enterprises mostly have no need for formalization and standardization [8]. Factors related to the external environment of organizations and relations with third parties also represent less important factors, which makes sense, since organizations find it more difficult to control areas that are influenced by external, often unmanageable factors [15]. Surprisingly, participating experts placed organizational culture and management maturity in the second half among less important factors, despite the fact that many authors believe that these aspects represent the key conditions for establishing efficient information security [12, 32, 35, 46]. Even though these two factors are not at the bottom of the scale, they are not considered as priorities in our model. The reason for this lies in the fact that even though the management of social and psychological aspects remains highly important, the high level of information security performance still relies primarily on high-quality technical and logical controls, adequate control over the use of confidential information and functioning of information systems. Security culture, which is defined as a system of norms, relationships, beliefs and behaviors developed by individuals with respect to organizational systems and information assets [55], is still strongly dependent on permissible practices, defined rules, regulations, rights and control measures. Security culture cannot be present and mature without the implementation of rules and sound development of tangible security measures. A similar finding applies to the state-of-play of security management, the maturity and competence of which is very important indeed, but depends on factors that are more difficult to control (such as adequate support by the management, financial capabilities, organizational structure). In addition, the security management’s function is of more strategic and social nature [41], since it is responsible for the adequate planning and monitoring of measures, providing compliance and formalization of rules, managing security culture or relationships and establishing links between information security and other organizational processes. Hence, its impact on information security performance is less obvious than the impact of practical short-term measures.

By analyzing the correlations between the factors and indicators, we were able to determine the most influential factors and define the type of impacts these factors have on one another. We found strong and statistically significant correlations in several areas. For example, measures of a physical, technical and logical nature, and security and information protection measures, are all positively correlated. In general, these areas fall within the scope of operational measures and have a similar purpose and technique, which makes their correlations reasonable. The fifth factor, which is related to information risks management, is the most influential of all factors. It is also associated with the second, third, fourth and tenth factor, which indicates that risk analysis and incident response depend on the skills in the field of technical security, protection of information resources, employee management and external environment control. Mutual correlations were also found between the third and fourth factor, the eighth and tenth factor and the ninth and tenth factor. Employee management affects the management of information resources and vice versa, while monitoring security trends in the environment is linked to security management and business relationships with third parties. By knowing these connections between individual areas, we can provide personalized guidance in selecting the initial steps for organizations under investigation. For example, if an organization finds that proper technical security controls are not in place, the management should focus primarily on the development of measures in this area, and then proceed with the development of the fifth, first and third factor, which have the most significant influence on technical capabilities.

The ISP 10×10M graphical presentation shows that it takes into account the complex and dynamic nature of organizational environment. It presupposes that information security performance is most strongly dependent on information risk management, information and employee control, as well as technical protection and physical security of information assets. Other areas have a lesser impact on the final result. For example, even though an organization invests maximum efforts to fully regulate all formal conditions of information security (e.g. compliance, relations with third parties) the final result will not be particularly high if it does not simultaneously guarantee specific operational security of the most important information assets. This means that the progression and combination of measures is vital for achieving a sound final result. The basic level of the ISP 10×10M contains the elementary and most important/critical information security measures. However, these only contribute to the fulfillment of the most general security needs. The intermediate level foresees that, apart from elementary measures, organizations also implement specialized measures aimed at specific organizational processes and develop information security as an important area of business. Organizations falling in the last, i.e. advanced performance level, apply the most demanding methods of protecting information and systems in terms of financial means and the necessary knowledge, which are conducted by specialized personnel and departments, and aimed at addressing clearly specified and most dangerous risks. Due to the complexity and specificity of measures, it is relatively difficult for organizations to reach the advanced level. In fact, this level covers those organizations that prove the most responsible and have the highest level of awareness in the field of security. By categorizing themselves into one of the described performance levels, organizations establish their starting points and identify which measures they must develop in order to advance into a higher performance level. In addition, organizations are able to determine which measures must be implemented immediately and which should be included into strategies and long-term plans. They could also compare themselves with other organizations, should the model be applied to a larger sample of organizations or to subsidiaries of a single organization.

The selected form of the ISP 10×10M, which puts operational conditions, control and repressive measures to the forefront and includes broader aspects of management, as well as tactical and strategic aspects in the final stages of establishing information security, is in line with the form of other existing models, such as the Information security maturity model [56], Security effectiveness framework study [19], CMMI for development [66] and Security operations maturity model [26]. The originality of this paper can thus be found in the fact that the research, which led to the development of the model, combines different factors and presents them in a single study.

Similarities with models referred to herein may be found in the distribution of security measures to the levels within the model, the weighting of criteria and application of different scales to perform the necessary measurements. The originality of the proposed model stems from the fact that it complements the technical aspects with organizational and management aspects. Experts often analyzed and examined such factors in isolation, i.e. separately from one another, while the proposed ISP 10×10M integrates them into a holistic assessment of information security performance. In addition, the weighting of indicators was also conducted in a different manner, i.e. independently of the target population, which is intended to apply the model. In the vast majority of existing models, the weighting process is performed arbitrarily (i.e. at the discretion of researchers) or by conducting research studies in target populations, where representatives of organizations are most often asked what they deem most important or requested to assess their own efficiency. Their answers are then used to measure the state-of-play with respect to individual measures, while the influence of an indicator is determined on the basis of the established difference between poorly and highly assessed indicators. Indicators that receive high marks are therefore deemed to have higher influence/impact and vice versa.

In the proposed setup of the model, however, the weighting of factors was conducted by relying on a group of experts, as described above. It must be stressed that this is the first documented method for conducting such research, i.e. for collecting data in the field of information security in Slovenia, which saw the meeting of the vast majority of experts, who stated their opinion regarding the significance of individual assessment criteria, in a single venue. When showing the preliminary results of their estimation, right after the research, most experts believed that the model layout is useful and accurate.

Despite the model’s general nature and universality, its content and methods can be adapted. For instance, many experts state that organizations have different security needs, which depend on certain specific factors. The level of technological dependence (i.e. informatization) is one of such factors, since those organizations that manage more complex information systems and greater quantities of information are allegedly more attractive for malicious perpetrators, more vulnerable and consequently more at risk than those that are technologically less developed [58]. In their empirical study authors observe that the need for security is directly proportional to the level of organizations’ informatization [67]. In addition, the impacts that security incidents have on organizations are also an important element. For example, organizations that process large quantities of confidential data and do their utmost to enable development and innovations may suffer more serious consequences due to the loss of data than smaller production plants or catering businesses for example [19]. Such requirements and factors can be incorporated into the proposed model, where additional indicators would be used to measure the level of technological dependency—informatization and impacts of incidents on business operations. In this context, greater dependency and stronger impacts of security incidents have a negative effect on the final result of information security performance. In order to meet the needs of individual organizations, the content of the ISP 10×10M can be adapted to specific organizational circumstances. Key performance indicators could be adapted by examining security strategies, security needs and conducting case analyses, while weights could be subsequently reorganized and factors’ content adjusted accordingly. Therefore, the model is flexible and dynamic, which is one of the main features of high-quality measuring instruments.

### Implications and Limitations

In terms of its practical application, the ISP 10×10M is useful for conducting case studies and also in-house evaluations (i.e. internal revisions). The management and security experts of an organization are responsible for taking decisions that contribute to the organization’s development, where the presented approach may be of great assistance. Those security managers, who understand the model, can use it as a tool to obtain information and adopt rational decisions. At the operational level, the ISP 10×10M is useful for IT staff drafting plans and identifying critical security areas since its application allows them to obtain answers to questions that cannot be answered by conducting isolated technical or economic analyses. Such questions may, for instance, include the following: How efficient is the organization? Is it efficient enough? How does it compare to other organizations? If there is a need for more reliable results, the model can be used in combination with other decision-making models (e.g. for establishing whether a certain measure recommended as a solution by the mode will, in fact, pay off). If an organization using the model finds that it is necessary to implement a new measure or improve the existing one, it can, for example, hereinafter use more complex performance-based earned value technique to measure technical performance for achieving planned functionality [68]. Moreover, since the ISP 10×10M is flexible, it can be fully harmonized with a specific security strategy by simply adapting individual criteria and weights. Its application is particularly appropriate for SMEs.

An example of the questionnaire devised for the ISP 10×10M evaluation purpose and its application in practice is presented in the supplement–S1 Questionnaire. By combining the questionnaire with the model layout and weighted variables presented in the paper one can perform a complete evaluation of the state-of-play with respect to information security in a specific or aggregated business case. The first part of the questionnaire (100 questions) is intended for internal evaluations or benchmarking with comparable cases, while its application for research purposes to larger samples in order to perform state evaluations should be combined with the demographic part of the questionnaire. In that case, it is recommended to normalize the obtained results with the level of informatization (i.e. the proportion of the sum of third and fourth questions with regard to their maximum score should be multiplied with the overall score) and to adjust the model’s critical limits accordingly (10–15:16–20 –basic level; 21–30:31–40 –intermediate level and 41–55; >55 advanced level). The idea is to consider the technological and security circumstances of organizations included in the research.

Despite of the practical application the herein presented ISP 10×10M has limitations as well. The main limitation of the model development process stems from a relatively small sample. The target group included experts, who are dealing with the management of information security professionally and on a daily basis. Since the development of the ISP 10×10M relied on measuring the importance of a vast quantity of variables, the sample should have been larger in order to meet general and formal statistical requirements. When considering the fact that the levels of professional public’ participation in research studies related to information security is very low and that it is almost impossible to compile a list of the entire population of experts, it was decided that an interactive group assessment of criteria is sufficient for setting the foundations of the model. However, any additional use of the model for market or scientific research would most certainly require a larger sample.

The ISP 10×10M is qualitative in nature and relies on personal evaluations. The final result depends on subjective assessments. In order to provide a higher validity of results, it is advisable for relevant areas to be evaluated by several individuals within an organization from board and management members to users, subcontractors, partners and persons responsible for security or IT. It is important for such evaluations to be provided by those, who are professionally acquainted with the state-of-play of organization’s information security system.

## Conclusion

The paper presents the theoretical basis and the process of developing the proposed model called ISP 10×10M, whose object of measurement is information security performance. The dependent variable is composed of 10 critical success factors, which are further measured by 10 different key performance indicators. Information security performance is therefore measured by 100 variables, each of which has its own weight. Once an organization completes the survey questionnaire, the final performance results are calculated. Subjects filling in the questionnaire must be familiar with the state-of-play within the organization and have some awareness of the issues, since they are requested to evaluate the extent to which the organization meets a certain criterion. After obtaining the final result, the organization is categorized into one of the six sub-levels and thus able to determine which measures need to be taken immediately and which should be adopted in a long-term perspective.

The process of devising the ISP 10×10M demonstrates that a high level of information security performance can only be achieved by adopting a multi-dimensional and multi-disciplinary approach. Organizations should first provide high-quality technological and physical protection, and then upgrade it by adequately managing social and user-related aspects, as well as by considering environmental and formal conditions in order to achieve maximum effects. In addition to the model itself, we presented the most important research studies, other existing methods for the provision of information security and relevant standards, which are fully in line with the findings of our research. By taking the present paper and its recommendations into account, organizations can find it much easier to adopt effective decisions and put the development of information security on the right track.

An important aspect of the paper presenting the ISP 10×10M model is related to its approach towards the construction and application of the model in practice. With the research conducted among information security experts and the model itself the paper’s implications are as follows:

- We demonstrated a unique development approach to a multilevel model for measuring information security performance.
- Information security measurement can be comprehensively defined by 10 CSFs and 100 KPIs.
- The presented model allows organizations to measure their capabilities and state-of-play in the field of information security solely by themselves.
- Information security performance is strongly influenced by operational and technical measures, which was taken into account in the model.
- The research showed that an organization’s capabilities in the field of information risk management system have the strongest impact on information security efficiency in comparison to other measured factors.
- Experts confirmed that the measurement of information security represents one of the measures necessary for improving the current state-of-play.

Further research could go in the direction of re-evaluation of the model composition and it’s variables. It would be useful to test these recommendations and measures, in the form of a Delphi method with international experts in the field. In case of conducting a re-study study we could also reduce the number of indicators and factors on the basis of calculated correlations, for formation of even more compact model with the same reliability. Since the model is already highly consistent, user-friendly and reliable, we should could focus primarily focus on testing the ISP 10×10M on a larger sample of organizations in order to assess the state-of-play of information security, and on conducting specific studies that will allow the identification of good and bad practices. We aim to create a database that will enable long-term monitoring of the state-of-play within the business environment and provide a better understanding of information security needs in the business sector.

## Supporting Information

S1 Questionnaire Questionnaire for organizations designed on the basis of ISP 10×10M. (DOCX)

S1 Table Synopsis of the key information security models and standards review. (DOCX)
