# Supplementary material for: Measuring Information Security Performance with 10 by 10 Model for Holistic State Evaluation
Source: PLoS One. 2016 Sep 21;11(9):e0163050. doi: 10.1371/journal.pone.0163050 (PMC5031431; doi:10.1371/journal.pone.0163050)
Supplement: S1 Questionnaire — (DOCX) [file pone.0163050.s001.docx]

**ISPM 10×10M – Questionnaire for Organizations Reference No.: _____**

Consider each of the areas listed below (from “P1” to “P10”) and assess to which extent your organization fulfills the criteria or to which extent it would be able to activate the listed measures in the event of a security incident. Please assess the criteria on the scale from 1 to 5: ***1- measures in this area have not been adopted yet, 2 – measures in this area are planned, 3 – measures in this area have been partially adopted, 4 – measures in this area are almost fully adopted, 5 – measures in this area are fully activated and functioning properly.*** Please fill in your assessments in the fields on the right-hand side of the table (“assessment”). Please assess all listed criteria – should you decide not to assess an individual criterion, we will presume that your organization is not fulfilling it and mark it with 1. Your answers will be entered into the formula of the model, which is used to calculate the efficiency of information security. If you are interested in obtaining an insight into the results (regarding your classification) and compare your efficiency with other participating organizations, please choose a reference number (any number) and enter it in the appropriate space in the top right-hand corner.

| **P1: Physical information security controls** | **Assessment** |
| --- | --- |
| 1. Fire, voltage and flood protection of buildings and premises |  |
| 1. Adequate installation and management of communication and power network |  |
| 1. Support systems for critical services – power supply, cooling, communication |  |
| 1. Control of third-party access to buildings and premises |  |
| 1. Control of employee access to buildings and premises |  |
| 1. Adequate installation and physical protection of hardware |  |
| 1. Regular maintenance of hardware |  |
| 1. Protection of ICT located outside organizations’ premises (MDM systems) |  |
| 1. Adequate building architecture and security plan in place – defined security areas |  |
| 1. Protection of buildings and premises against break-ins and wiretapping |  |
| **P2: Technical and logical security controls** | **Assessment** |
| 1. Malware protection |  |
| 1. Logical security of programs, systems and databases – identification/authorization |  |
| 1. Technical protection of local networks (LAN) and network devices |  |
| 1. Technical measures aimed at protecting information during their storage |  |
| 1. Technical measures aimed at protecting communications and information during transfer |  |
| 1. Access control – log management, activity monitoring |  |
| 1. Adequate system capabilities and capacities for information processing – system reliability |  |
| 1. Standardization of workstations |  |
| 1. Change management – analyses of impacts that technology changes have on existing systems |  |
| 1. Regular (automatic) security updates of software and systems |  |
| **P3: Information resources management** | **Assessment** |
| 1. Security categorization of information |  |
| 1. Defined administration and other responsibilities related to information management |  |
| 1. User guidelines for handling information |  |
| 1. Implementation of the “need-to-know” principle |  |
| 1. Control over the exercise of administrator and system rights |  |
| 1. Definition and protection of organization’s intellectual property |  |
| 1. Definition and protection of personal data |  |
| 1. Provision of data processing traceability – audit trails |  |
| 1. Adequate deletion of data, destruction of equipment and physical documentation |  |
| 1. Information archiving and regular back-ups |  |

| **P4: Employee management** | **Assessment** |
| --- | --- |
| 1. Raising employees’ awareness regarding information risks and policies |  |
| 1. Defined user responsibilities related to the use of confidential systems and data |  |
| 1. Defined disciplinary proceedings, sanctions and infringement proceedings |  |
| 1. User rights management throughout employment – before, during, after employment |  |
| 1. Security vetting of employees |  |
| 1. Employee agreements and declarations concerning the protection of confidentiality |  |
| 1. Provision of technical and consultative support to employees |  |
| 1. Defined remote access and teleworking procedures |  |
| 1. Protection of employee rights during information security control procedures – protection of privacy |  |
| 1. Professional training of security and technical personnel |  |
| **P5: Information risk management and incident handling** | **Assessment** |
| 1. Business continuity plan and policy |  |
| 1. Automated early warning systems – IDS, IPS, SIEM |  |
| 1. Defined procedures for reporting and handling detected irregularities |  |
| 1. Crisis management – plans for responding to critical security risks |  |
| 1. An alternative location (i.e. hot spot) for the most important parts of information systems |  |
| 1. Incident monitoring, recording and analysis – experiential learning |  |
| 1. Forensic procedures and evidence gathering for incident investigations |  |
| 1. Information risk management – analysis and evaluation |  |
| 1. Analyses of former information incidents’ impacts on business operation – damage assessment |  |
| 1. Assessment of existing security controls’ efficiency – performance measurement |  |
| **P6: Organizational culture and top management support** | **Assessment** |
| 1. Ethical, socially responsible and transparent security management |  |
| 1. Pursuing the principle of efficiency in information security – economy/cost optimization |  |
| 1. Good relations and constructive debates regarding security controls between organizational departments |  |
| 1. Inclusion of information security in the planning of organizational projects and changes |  |
| 1. Leadership familiarity with security needs – direct communication channels |  |
| 1. Users’ general satisfaction and confidence with respect to information security |  |
| 1. Organizations’ innovativeness, excellence and continuous development in the field of information technology |  |
| 1. Adequate staffing and financial support to information security |  |
| 1. Clearly defined organizational hierarchy and job classification regarding management of organizational security |  |
| 1. Leadership involvement in information security planning |  |
| **P7: Information security policy and compliance** | **Assessment** |
| 1. Adoption of a formal information security policy |  |
| 1. Policy’s breakdown into sub-areas and orderly documentation |  |
| 1. Monitoring the respect of policies among users during their everyday work |  |
| 1. Compliance with international standards and recommendations |  |
| 1. Continuous development and upgrading of information security – control of risks and conformity |  |
| 1. Regular management reviews and internal audits |  |
| 1. Compliance with relevant legislation |  |
| 1. Fulfillment of contractual security obligations |  |
| 1. Use of licensed products and services |  |
| 1. Analysis of examples of information security best practices – benchmarking |  |

| **P8: Security management maturity** | **Assessment** |
| --- | --- |
| 1. Strategic and long-term planning of information security – proactive approach |  |
| 1. Development of information security as a business function or special department/ 2. service within an organization |  |
| 1. Adequate personnel structure – recruitment of qualified staff |  |
| 1. Formal authority of security personnel – ability of decision-making |  |
| 1. Division between system-related and security tasks – separation between IT and security division |  |
| 1. Cooperation with other organizational authorities in information security planning |  |
| 1. Regular vertical and horizontal security meetings |  |
| 1. Team decision-making regarding management of critical security risks |  |
| 1. Management of employees’ security culture and motivational activities |  |
| 1. Legitimacy of information security – compliance with user requirements |  |
| **P9: Third-party relationships** | **Assessment** |
| 1. Formalized contractual relationships with partners and suppliers regarding information security |  |
| 1. Defined security responsibilities with respect to customers |  |
| 1. Involvement of third parties in the implementation of information security measures |  |
| 1. Good customer relations – building trust and reputation/organizations’ goodwill |  |
| 1. Testing ICT before acquisition – defined acceptability criteria and quality |  |
| 1. Security vetting of business partners and suppliers |  |
| 1. Defined and regulated security of e-business |  |
| 1. Adequate technical protection of inter – organizational information systems |  |
| 1. Formalized contractual relationships for the processing and exchange of personal data |  |
| 1. Liability insurance covering information security events and incidents |  |
| **P10: External environment connections** | **Assessment** |
| 1. Flexibility of organizations – adapting to changes in the sector and the environment |  |
| 1. Successful management of competitive and external pressures |  |
| 1. Cooperation with other sectoral organizations – inter-organizational strategic security ties |  |
| 1. Participation in economic and business associations, societies and groups |  |
| 1. Cooperation with competent authorities when dealing with information incidents |  |
| 1. Cooperation with security consultant groups and external audits of information security |  |
| 1. Active participation in foreign/international environments – international cooperation for knowledge sharing |  |
| 1. Defined rules governing communication with the public and competitive organizations |  |
| 1. Monitoring technological developments and implementing innovations regularly |  |
| 1. Monitoring and analyzing security trends – development of threats and vulnerabilities |  |

Since the data are anonymous (no information pertaining to the identity of your organization is collected), you are kindly asked to answer the following questions, which will help us to classify the participating organizations into groups.

1. **On the scale from 1 to 5, please assess the level of your organization’s dependency on IT (1 – very high, 5 – very low):**

| 1 | 2 | 3 | 4 | 5 |
| --- | --- | --- | --- | --- |

1. **On the scale from 1 to 5, please assess the impact an information incident (loss or disclosure of confidential information) would have on your organization’s reputation (1 – very high, 5 – very low).**

| 1 | 2 | 3 | 4 | 5 |
| --- | --- | --- | --- | --- |

1. **Please indicate the number of employees in your company** (data is collected in order to assess the size: ________ employees
2. **Please give an approximate estimation of profit per employee in your organization:** ___________ /employee
3. **Your organization (please circle the correct answer):**

- operates only in domestic/origin country.
- operates in domestic country and abroad.

1. **Your organization (please circle the correct answer**):

- Is a part of public (state) sector.
- Is a part of private sector.

1. **Please name the main business activity of your organization**: _____________________
