# Supplementary material for: Measuring Information Security Performance with 10 by 10 Model for Holistic State Evaluation
Source: PLoS One. 2016 Sep 21;11(9):e0163050. doi: 10.1371/journal.pone.0163050 (PMC5031431; doi:10.1371/journal.pone.0163050)
Supplement: S1 Table — (DOCX) [file pone.0163050.s002.docx]

| **Publication** | **Measurement and security controls recommendations** |
| --- | --- |
| **NIST SP 800 – 55 Rev. 1** Performance Measurement Guide for Information Security: NIST, 2008, and **NIST** **SP 800 – 53 Rev. 4** Security and Privacy Controls for Federal Information Systems and Organizations [1, 2] | Special Publications from the 800 series provide recommendations for regulating different areas related to computer security. Two guides are particularly important for the identification of critical controls and measurement. The SP 800-55 Rev. 1 (2008) defines conditions for sound measuring procedures, which include: defining stakeholders, goals and objectives; policy and procedures review; type of measurement selection; identification of performance criteria; feedback and measurement life cycle. Together with the SP 800-53 Rev 4, which clearly defines and describes information security controls, the aforementioned guide recommends that measurements for establishing its performance be conducted in the following areas: (1) security budget; (2) vulnerability management; (3) access control; (4) awareness and training; (5) audit and accountability; (6) certification, accreditation and security assessment; (7) configuration management; (8) contingency planning; (9) identification and authentication; (10) incident response; (11) maintenance; (12) media protection; (13) physical and environmental security; (14) planning; (15) personnel security; (16) risk assessment; (17) system and services acquisition; (18) system and communication protection; (19) system and information integrity. |
| **ISO/IEC 27001:2013 and ISO/IEC 27002:2013** [3, 4] | The ISO/IEC 27001: 2013 (Information security management system – requirements) and ISO/IEC 27002: 2013 (Code of practice for information security management) international standards define the ISMS model, security controls, guidelines for their implementation and best practice recommendations for establishing and governing information security in organizations. The model contains the following main topics and categories: (1) management guidelines and information security policies; (2) organization of information security; (3) human resource security; (4) asset management; (5) access control; (6) cryptography; (7) physical and environmental security; (8) operations security; (9) communications security; (10) system acquisition, development and maintenance; (11) supplier relationships; (12) information security incident management; (13) information security aspects of business continuity; (14) compliance. |
| **Critical security controls (CSC):** SANS Institute, 2016 [5] | Critical security controls contain a list of security functions and recommendations to counter advanced and sophisticated information threats developed in cooperation with the Council of Cybersecurity. Version 6 of the CSC provides twenty recommendations: ([1) inventory of authorized and unauthorized devices;](http://www.sans.org/critical-security-controls/control/1) ([2) inventory of authorized and unauthorized software;](http://www.sans.org/critical-security-controls/control/2) ([3) secure configurations for hardware and software on mobile devices, laptops, workstations and servers;](http://www.sans.org/critical-security-controls/control/3) ([4) continuous vulnerability assessment and remediation;](http://www.sans.org/critical-security-controls/control/4) ([5) controlled use of administrative privileges;](http://www.sans.org/critical-security-controls/control/5) (6) [maintenance, monitoring, and analysis of audit logs;](http://www.sans.org/critical-security-controls/control/6) (7) [email and web browser protections;](http://www.sans.org/critical-security-controls/control/7) ([8) malware defenses;](http://www.sans.org/critical-security-controls/control/8) ([9) limitation and control of network ports, protocols, and services](http://www.sans.org/critical-security-controls/control/9); ([10) data recovery capability;](http://www.sans.org/critical-security-controls/control/10) ([11) secure configurations for network devices such as firewall routers, and switches;](http://www.sans.org/critical-security-controls/control/11) ([12) boundary defense;](http://www.sans.org/critical-security-controls/control/12) ([13) data protection](http://www.sans.org/critical-security-controls/control/13); ([14) controlled access based on the need to know](http://www.sans.org/critical-security-controls/control/14); ([15) wireless access control;](http://www.sans.org/critical-security-controls/control/15) ([16) account monitoring and control;](http://www.sans.org/critical-security-controls/control/16) ([17) security skills assessment and appropriate training to fill gaps](http://www.sans.org/critical-security-controls/control/17); ([18) application software security](http://www.sans.org/critical-security-controls/control/18)([19) incident response and management;](http://www.sans.org/critical-security-controls/control/19) ([20) penetration tests and red team exercises.](http://www.sans.org/critical-security-controls/control/20) |
| **COBIT 5 for information security** [6] | ISACA’s COBIT 5 business framework for information security includes the following main principles: (1) meeting stakeholders’ needs; (2) covering enterprise end-to-end; (3) applying a single integrated framework; (4) enabling a holistic approach; (5) separating governance from management. In addition, the COBIT 5 main control areas include: (1) risk management; (2) access control; (3) event detection and response; (4) system acquisition and development; (6) operations and procedures; (6) disaster recovery; (7) external parties; (8) regulatory compliance. The COBIT Maturity Model used to measure organizations’ efficiency in managing information security is composed of six maturity levels. The model presupposes that information security may be (1) non-existent (2) ad hoc; (3) repeatable but intuitive; (4) based on defined process; (5) managed and measurable; (6) optimized. |
| **Business model for information security (BMIS)** [7] | ISACA’s three-dimensional business model for managing information foresees that information security must develop at four key levels: (1) organizations’ design and strategy; (2) people; (3) processes; (4) technology. The BMIS also defines six dynamic interconnections between people, technology, organizations and processes: (1) governing; (2) culture; (3) enabling and support; (4) emergence; (5) human factors; (6) architecture. The most relevant components and procedures of a security system defined in the BMIS include: (1) enterprise structure; (2) organizations’ security structure; (3) authentication, authorization, detection; (4) employee procedures; (5) account procedures; (6) department procedures; (7) audit procedures; (8) reporting procedures; (9) incident response procedures; (10) policy, standards; (11) awareness programs; (12) application system design. The model also emphasizes the following security measures and solutions: ID management, employee monitoring, VPN, emergency changes, CCTV, switching, firewalls, encryption, virtualization, forensic analysis, HIDS/NIDS, DPL and ICT continuity software. |
| **PAS 555 Cyber security risk – Governance and management – specification** [8] | **The PAS 555 business framework for effective cyber security in enterprises focuses on technical, physical, cultural and behavioral aspects of security, including leadership and governance. The main security topics and control groups include:** (1) commitment to a cyber security culture; (2) security context; (3) business architecture strategy; (4) capability development strategy; (5) supplier and partner strategy; (6) technology strategy; (7) business resilience; (8) compliance with legislation and other standards; (9) risk assessment; (10) protection and mitigation; (11) detection and response; (12) recovery; (13) compliance analysis and continual improvement. |
| **10 steps to cybersecurity** [9] | The cybersecurity guide provides recommendations to organizations about the necessary steps for the most effective information security investment. The main topics of the document include: (1) home and mobile working (policy and training, data protection); (2) user education and awareness (rules of acceptable use of the IS, training programs); (3) incident management (incident response and disaster recovery, planning and testing, special training programs for management, report to law enforcement); (4) information risk management regime (policies, management and leadership support, risk assessment and defined acceptable risks); (5) managing user privileges (account management processes, limitation of accounts and privileges, monitoring user activity, access controls, audit logs); (6) removable media controls (all access to removable media control, limitations of media types, media scans); (7) monitoring (monitoring strategy and policies, network monitoring, log analysis, unusual activity monitoring); (8) network security (internal and external network control, network perimeter management, filtering, security control testing); (9) malware protection (software, policy and scanning); (10) configuration (security patches, system inventory, baseline build for all devices). |
| **IASME (The standard for information assurance for small and medium-sized enterprises)** [10] | According to the recommendations of the IASME standard, investments in cyber security should fall into three categories where their main performance elements and controls are defined as follows:  (a) Protection - (1) organization (leadership commitment, employment of security professionals, coordination group, expert advice on threat landscape); (2) risk assessment (assessing information risks, determining risk appetite and managing threats); (3) policy and compliance (identification of organization’s legal, statutory, regulatory and contractual obligations, providing management direction and support, preventing loss, destruction and falsification of information systems and information with legal obligations); (4) assets (record of key organizational assets, ensuring information security while using cloud assets, social media and personal mobile computing, safe disposal of assets); (5) people (security checks before employment, security awareness programs, user privilege management before, during and after employment); (6) physical and environmental protection (prevention of unauthorized access, loss, damage, theft of information and assets); (7) operations and management (patching, security of electronic commerce services and service delivery with third parties); (8) access control (preventing unauthorized access to information, applications, facilities, network, system files and source code);  (b) Detection - (9) malware and technical intrusion (detecting and protecting systems and information from malicious software); (10) monitoring (detection of unauthorized information processing by audit and accounting services);  (c) Recovery - (11) backup and restore (performance testing, secure double location); (12) incident management (identification of security events and weaknesses, defined responsibilities and procedures, audit trails, threat containment and incident analysis, reporting, learning); (13) disaster recovery/business continuity (identification of critical vulnerabilities and processes, organizational plans to counteract and recover from loss of integrity and availability, plan testing). |
| **Governing for enterprise security – (GES)** [11] | The GES is intended for business leaders as a guide for implementing an effective program to govern information technology and information security. Guidelines for IT and security governance describe eleven characteristics of effective security in the form of the following statements regarding information security: (1) an enterprise-wide issue; (2) leaders are accountable; (3) viewed as business requirement; (4) risk-based; (5) defined roles, responsibilities and segregation of duties; (6) addressed and enforced within a policy; (7) commitment of adequate resources; (8) staff are aware and trained; (9) development life cycle requirement; (10) planned, managed, measurable and measured; (11) reviewed and audited. |
| **Security effectiveness framework study** [12] | The framework explains key components of an effective security operation that helps to deliver a measurable impact on business performance. In the study, security experts identified six critical metrics for achieving security effectiveness: (1) uptime; (2) compliance; (3) threat containment; (4) cost management; (5) breach prevention and (6) policy enforcement. |

**References**

1. National institute for standards and technology [NIST]. NIST SP 800-55 Rev.1. Performance measurement guide for information security. Gaithersburg, MD: NIST; 2008.
2. National institute for standards and technology [NIST]. NIST SP 800-53A Rev.4. Assessing security and privacy controls in federal information systems and organizations: Building effective security assessment plans. Gaithersburg, MD: NIST; 2013.
3. International Organization for Standardization [ISO], International Electrotechnical Commission [IEC]. ISO/IEC 27001: 2013. Information technology - Security techniques - Information security management systems – Requirements. Geneva:ISO/IEC; 2013.
4. International Organization for Standardization [ISO], International Electrotechnical Commission [IEC]. ISO/IEC 27002: 2013. Information technology - Security techniques - Code of practice for information security controls. Geneva: ISO/IEC; 2013.
5. The Center for Internet security.critical security controls for effective cyber defense, version 6.0; 2015.Available from <https://www.sans.org/critical-security-controls>
6. ISACA. COBIT 5 for information security. Control Objectives for Information and Related Technology: A business framework for the governance and management of enterprise IT. Rolling Meadows, IL: ISACA; 2012.
7. ISACA. Business model for information security [BMIS]; 2009. Available from: <http://www.isaca.org/Knowledge-Center/BMIS/Pages/Business-Model-for-Information-Security.aspx>
8. National Standards Body [BSI]. PAS 555: 2013. Cyber security risk. Governance and management. Specification. London: BSI; 2013.
9. Government Communications Headquarters [GCHQ, The Department for business innovation & skills [BIS], Centre for the protection of national infrastructure [CPNI]. 10 steps to cyber security: Cyber security guidance for business; 2012. Available from: <https://www.gov.uk/government/publications/cyber-risk-management-a-board-level-responsibility>
10. IASME. The standard for Information assurance for small and medium sized enterprises, Issue 2.3. Malvern: IASME Consortium; 2013.
11. Allen JH, Westby JR. Governing for enterprise security: Implementation guide. Article 1 - Characteristics of effective security governance. Pittsburgh, PA: Carnegie Mellon University; 2007. Available from: <http://resources.sei.cmu.edu/asset_files/WhitePaper/2007_019_001_54375.pdf>
12. Ponemon Institute. Security effectiveness framework study; 2010. Available from: <http://trionlogics.com/wp-content/uploads/Security-Effectiveness-Framework-Study.pdf>
